# Supplementary figures and images for: Apremilast Improves Endothelial Glycocalyx Integrity, Vascular and Left Ventricular Myocardial Function in Psoriasis
Source: Pharmaceuticals (Basel). 2022 Jan 30;15(2):172. doi: 10.3390/ph15020172 (PMC8876564; doi:10.3390/ph15020172)

**Figure S1.** CONSORT 2010 flow diagram.

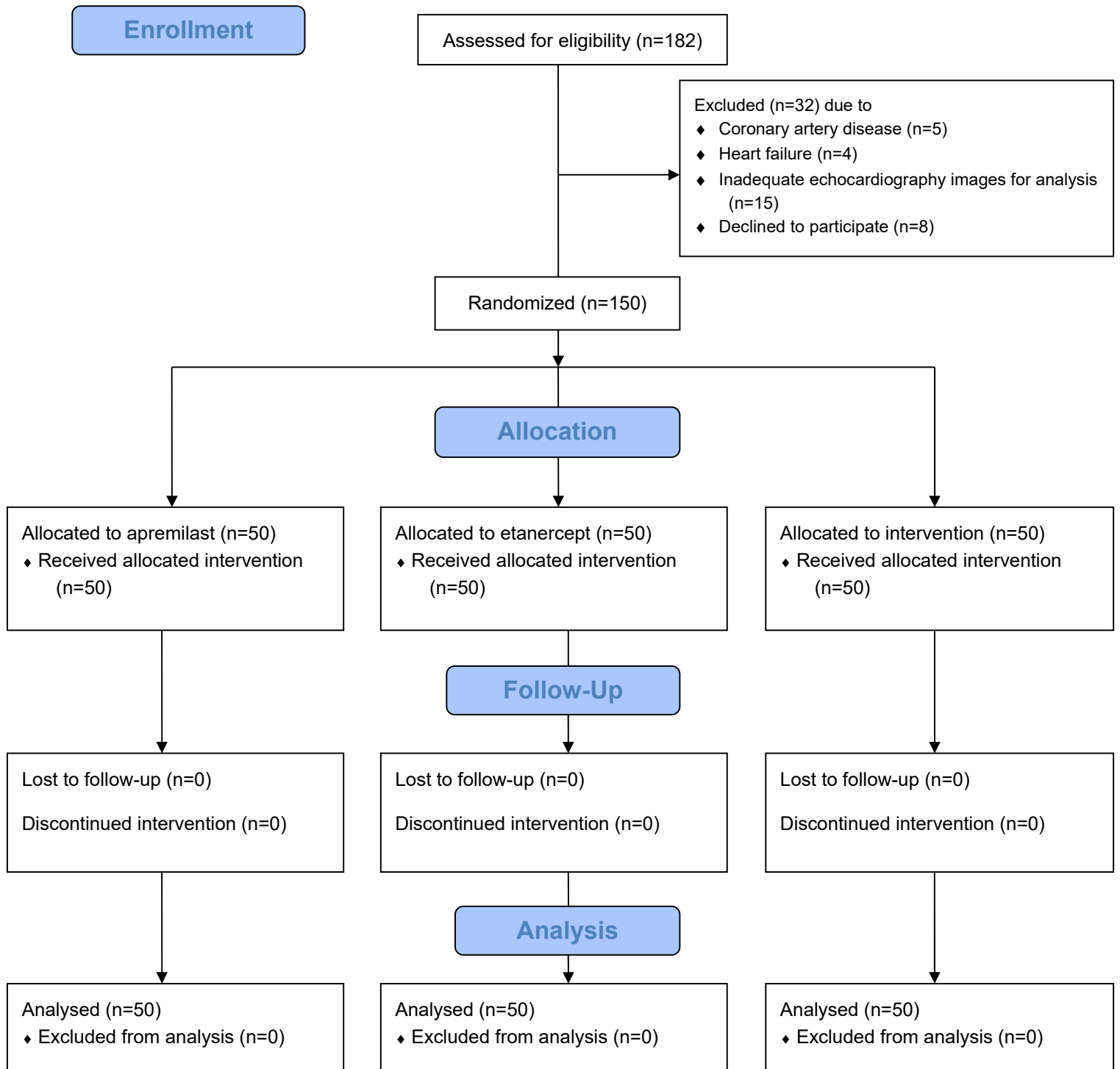

Supplement: Supplementary file 1 [file pharmaceuticals-15-00172-s001.zip › pharmaceuticals-1530594-supplementary.pdf]
